# Recycling of Polyurethane Waste: Facile Hydrothermal Conversion Using Acidic and Basic Additives

**DOI:** 10.1002/cssc.202502372

**Published:** 2026-01-05

**Authors:** Hongqi Wang, Himanshu Gupta, N. Raveendran Shiju

**Affiliations:** ^1^ Catalysis Engineering Group Van ‘t Hoff Institute for Molecular Sciences University of Amsterdam Amsterdam Netherlands

**Keywords:** base, catalyst, hydrolysis, hydrothermal liquefaction, polyurethane waste

## Abstract

Polyurethane (PU) is a widely utilised plastic material due to its versatile properties. The chemical recycling, especially by hydrothermal treatment, is an effective way to achieve the circular use of PU. This article reports the results of hydrothermal treatment of PU with and without the use of acidic and basic catalysts. Both non‐catalytic and catalytic approaches showed that PU could be depolymerised to the monomers using hydrothermal treatment. The use of a catalyst improved PU conversion and 2,4‐toluenediamine (TDA) yield. An organic amine showed better catalytic activity than inorganic base NaOH, inorganic acid H_2_SO_4_, and organic acid acetic acid. Among the catalysts tested, the organic amine ethylenediamine exhibited the highest activity, achieving a TDA yield of 13.6 wt% and a PU conversion of 28.2% at 180°C. Organic bases outperformed inorganic acids and bases, such as H_2_SO_4_, acetic acid, and NaOH, which is attributed to their ability to form ionic interactions with PU‐derived zwitterions and their uniform distribution across vapour and liquid phases under vapour–liquid equilibrium.

## Graphical abstract

Ethylenediamine efficiently catalyses the hydrothermal recycling of polyurethane (PU) into monomers. The N—H functional groups promote carbamic acid decomposition via zwitterion interactions, outperforming inorganic acids and bases, and enabling selective depolymerisation of PU under mild conditions for circular chemical utilisation.

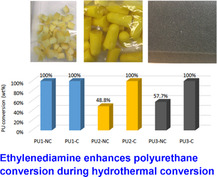

## 1 Introduction

In recent years, plastics waste management has become a major global concern, driving increasing research into chemical recycling and circular economy strategies to mitigate environmental impacts. Advanced conversion processes such as hydrothermal liquefaction (HTL) and pyrolysis have shown promise for transforming mixed or polyolefin‐rich plastics waste into valuable hydrocarbons and reusable chemical feedstocks, thereby reducing dependence on virgin fossil resources and enabling material circularity [1, 2, 3–4]. Beyond polyolefins, chemical recycling approaches are also being extended to condensation polymers, such as polyethylene terephthalate (PET), where catalytic depolymerisation methods like glycolysis have demonstrated efficient recovery of monomers for reuse in new materials [5, 6–7]. Similarly, emerging strategies are addressing the recycling and reuse of specialised polymer systems such as olefinic battery separators and thermoset plastics, expanding the scope of circular technologies to more complex waste streams [8].

Polyurethane (PU) is a common and versatile polymer widely used in our society due to its durability and low cost, such as flexible foam in furniture and rigid foam in automotive interiors [9, 10]. PU can be tailored into thermoplastic or thermoset forms, depending on the polyols and polyisocyanates employed in the production process [11]. The urethane group (–HN–COO–) is the typical functional group in PU, irrespective of the utilised polyols and polyisocyanates. The recycling of PU waste is not only crucial for alleviating the environmental burden associated with the current disposal method of landfilling and but also for recovering valuable feedstocks in a sustainable and circular manner.

PU waste could be recycled by both mechanical and chemical methods [12]. The physical treatment in mechanical recycling requires a high level of purity and may cause the degradation of product quality [13]. The chemical recycling includes glycolysis, hydrolysis and pyrolysis [9]. Glycolysis refers to the depolymerisation of PU through transesterification reactions with a glycol, typically conducted under elevated temperature and pressure conditions when using glycols such as ethylene or propylene glycol. However, the polyol might undergo side reactions with the presence of a catalyst at high temperature. Similar to glycolysis, PU could be converted into polyol and amine intermediates by hydrolysis [14]. The pyrolysis of PU in the absence of oxygen environment could produce gas and complex oil at high temperature.

The liquefaction process is a viable route for converting organic waste into valuable oil products, offering a milder and energy‐efficient alternative for chemical recycling under environmentally benign conditions [15]. HTL, especially, is one of the most promising and practical recycling approaches because water is a cheap, green reactant and solvent and because the reaction conditions are milder than those required for pyrolysis [16]. The PU form could be decomposed into diamine and polyols by reacting with superheated steam at 232–316°C and atmospheric pressure [17]. The hydrolysis could reduce the volume of low‐density PU by a factor of 30 at 200°C, leading to the formation of an upper aqueous phase containing toluene diamine and a lower phase of polyols [18].

Even though there have been some investigations about the conventional HTL of PU, the catalytic HTL of PU waste is still limited. It has been reported that KOH, when used as a base catalyst, does not markedly influence the overall product mass balance but enhances hydrolysis by promoting the formation of a larger number of smaller molecular compounds compared to non‐catalytic HTL at 350°C. This enhancement is likely due to the generation of NH_3_ during the process, which may further facilitate hydrolysis [19, 20]. Dai and co‐authors investigated the HTL of PU from 150 to 350°C and the optimal hydrolysis condition was determined to be 250°C and 30 min [21]. Similarly, the authors also reported that the addition of bases such as NaOH and diaminotoluene only improved the yield of 2,4‐toluenediamine (TDA) slightly and did not lower the activation energy [21]. However, the detailed mechanism is still not clear, especially the catalytic process during the hydrothermal decomposition of PU. It is well known that H^+^ or OH^−^ could be produced in the sub‐ or supercritical state of water, which can function as a catalyst to the decomposition reaction.

In this study, we have used various inorganic and organic acid and base catalysts (e.g. NaOH, ethylenediamine, H_2_SO_4_ and acetic acid) for the HTL of the PU waste from mattresses and we found that an organic base ethylenediamine, can catalyse and initiate the hydrolysis of PU effectively. Compared to the HTL at harsh conditions, the ethylenediamine catalysed HTL could achieve higher selectivity to TDA at low temperature. HTL at different temperatures and reaction times was also conducted to fully convert the PU waste. In addition, different PU wastes (e.g. from earplug and packaging foam) were also hydrothermally liquefied with and without catalysts to validate the concept.

## 2 Experimental

### 2.1 Materials

The waste PU samples were from a mattress (labelled as PU1), an ear plug (labelled as PU2), and from packaging foam (labelled as PU3). All PU samples were sourced from waste. The thermogravimetric analysis (TGA) and Fourier‐transform infrared spectroscopy (FT‐IR) characterisation of the PU samples are in Figures S1 and S2 (supporting information). The C—N at 1592 cm^−1^ and the C—O—C bond at 1220 cm^−1^ are the target bonds to be cracked.

Tetrahydrofuran (THF, ≥99.0%), ethylenediamine (≥99%), chloroform‐d (99.8 atom% D), diethylamine (99.5%), benzylamine (99%), dibenzylamine (97%), acetic acid (100%), and sulphuric acid (95–97%) were purchased from Sigma–Aldrich and used as they are without further treatment. Acetone (≥99%) was bought from VWR.

### 2.2 Experimental Set‐up

The HTL of PU was conducted in a stainless‐steel autoclave (Parr model: 4560; volume: 450 mL) with a 4848 controller. The PU foam was cut into 1 × 1 × 1 cm cube for easy handling and loading. Typically, 4 g PU foam and 100 g H_2_O together with 0.2 g catalyst were loaded into the reactor. 4 g is the maximum loaded amount for PU1 due to the low density. The reactor was flushed three times using argon and subsequently depressurised to atmospheric pressure. After heating up to the desired temperature (e.g. 180 or 210°C) with the stirring speed being 400 rpm, the temperature was kept for a certain amount of time (e.g. 30 min). Then, the reactor was cooled down immediately with an ice‐water bath after removing the heating mantle. After cooling, the gas was collected into a gas bag to be analysed by a gas chromatography (GC)‐flame ionization detector (FID)/thermal conductivity detector (TCD). The liquid and solid products were collected and separated by centrifuge, and subsequently analysed gravimetrically for mass balance closure calculation and further analysis. The unconverted paste or solid phase products were dried at 120°C overnight.

### 2.3 Definitions

The definitions are as follows:

(1) $$\text{conversion}=\frac{\text{reactant}\mathrm{PU}-\text{remained solid}\mathrm{or}\text{paste}}{\text{reactant}\mathrm{PU}}\times 100\mathrm{wt}\%$$

where the reactant PU means feedstock PU.

(2) $$\text{phase yield}=\frac{\text{weight}\mathrm{of}\text{liquid}\mathrm{or}\text{solid}\mathrm{or}\mathrm{gas}}{\text{weight}\mathrm{of}\text{reactant }\mathrm{PU}+H_{2}O}\times 100\mathrm{wt}\%$$

(3) $$\mathrm{TDA}\text{yield}=\frac{\text{weight}\mathrm{of}\mathrm{TDA}\mathrm{in}\text{liquid}\mathrm{and}\text{solid phase}}{\text{weight}\mathrm{of}\text{reactant}\mathrm{PU}}\times 100\mathrm{wt}\%$$

The conversion of PU in Equation (1) might be lower than the real value because the remained paste or solid phase may also contain the cracked polymer, probably in the form of oligomer and monomer. The phase yield in Equation (2) is based on the total weight of PU and water because the products are distributed in both phases. The Equation (2) is useful for mass‐balance analysis across the entire reactor system. The TDA yield in Equation (3) is based on the reactant PU.

### 2.4 Characterisation

The TGA was analysed using a TGA550 (TA Instruments). After stabilising at 40°C for 5 min, the PU samples were heated to 700°C at 10°C/min and then maintained for 10 min in 50 mL/min N_2_.

The GC‐mass spectrometry (GC–MS) was analysed using Agilent 5977 GC/MSD. The columns include a guard column/restriction gap CP802505 (1.5 m × 150 μm) and a HP‐5 ms capillary column (32 m × 250 μm × 0.25 μm). The injection volume is 1 μL and the split ratio is 20:1. After holding at 60°C for 3.5 min, the oven was heated from 60 to 250°C at 30°C/min and then held for another 6 min. The gas phase product was analysed using a GC‐FID/TCD, with a carboxal 1010 column (15 m × 0.32 mm) and a RT—Q bond column (3 m × 0.32 mm) for TCD and a Rt‐Alox/Na2SO4 column (10 m × 0.32 mm) and a wax column (4 m × 0.32 mm). The TDA in the product was quantified by using standard compounds to establish a calibration line between TDA concentration (wt%) and the area of extracted ion chromatograms (ion = 122) as shown in Figure S3 (supporting information).

FT‐IR was acquired from 400 to 4000 cm^−1^ using a Bruker Platinum‐ATR with the resolution being 4 cm^−1^ and sample scan time being 32. Nuclear magnetic resonance (NMR) was acquired by a Bruker Avance 300 MHz NMR spectrometer using CCl_3_D as the solvent.

## 3 Results and Discussion

### 3.1 Effect of Different Catalysts on the HTL of PU

#### 3.1.1 Proposed Mechanism

The depolymerisation of PU during HTL mainly involves the cracking of C—N and C—O bonds of the urethane groups (–HN–COO–). The H bond among the base catalyst, H_2_O molecules and PU polymers may affect the preference for the cleavage of these two types of bonds in the PU. At higher temperatures, the H bonds of H_2_O become weaker even though its ability to dissolve organic molecules increases [22]. It is highly possible that the organic base catalyst will first attach to the polymer chain with the help of a hydrogen bond in a way:

(4) $$\mathrm{PU}-C=O\cdot \cdot \cdot H-O^{-}\left(\text{base}\right)$$

(5) PU−N−H· · ·N−H(amine)

(6) PU−C=O· · ·H−N(amine)

which could cause a different cracking path of the urethane group in the aqueous environment. The dotted line (···) in Equations (4)–(6) represents the hydrogen bond between the oxygen/nitrogen atom and the hydrogen atom. The increase of intermolecular H bond between polymer and organic base/H_2_O could accelerate the hydrolysis [23].

**FIGURE 1:**
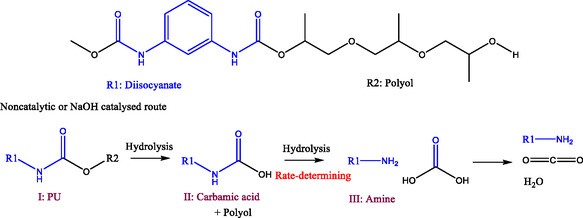
Proposed reaction route of PU during non‐catalytic or NaOH‐catalysed HTL. Substrate PU (I) undergoes hydrolysis to produce carbamic acid (II) and polyol. Under hydrothermal conditions, transient isocyanate species may form but are immediately hydrolysed to carbamic acid. Only the dominant aqueous pathway is illustrated. Carbamic acid (II) further undergoes hydrolysis to form amine, CO_2_ and H_2_O which is the rate‐determining step.

The proposed reaction route for the non‐catalytic or NaOH‐catalysed hydrothermal treatment of PU is shown in Figure 1. It is reported that the polyols are formed before amines during the hydrolysis of PU [24]. We propose that the ester group in the urethane group is the most easily cracked, whereas the cracking of the C—N bond is the rate‐determining step during the non‐catalytic or NaOH‐catalysed route. The PU (I) is first cracked into a carbamic acid (II) and polyol by hydrolysis, and the subsequent hydrolysis would produce amine (III) and CO_2_. Although the formation of isocyanate intermediates cannot be completely ruled out, such species are highly unstable in water and rapidly hydrolyse to carbamic acid, which then decomposes into amines and CO_2_. Therefore, the pathway shown in Figure 1 represents the dominant hydrothermal route.

**FIGURE 2:**
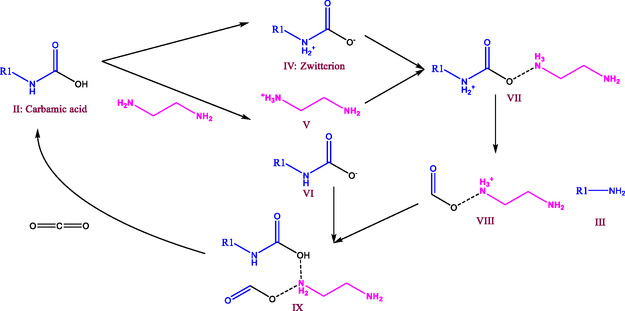
Ethylenediamine catalysed hydrolysis to crack the C—N bond during HTL of PU. The carbamic acid (II) undergoes tautomerism to form a zwitterion (IV) and acid‐based reaction with ethylenediamine to produce two ions (V and VI). Then the zwitterion (IV) and cation (V) derived from ethylenediamine react to form an amine (III) and a cation (VIII). The subsequent ion reaction between (VI) and (VIII) would produce CO_2_ and ethylenediamine.

The ethylenediamine‐catalysed HTL route is proposed as in Figure 2. The carbamic acid (II) undergoes tautomerism to form a zwitterion (IV) and acid‐based reaction with ethylenediamine to produce two ions (cation: V and anion: VI) [25]. Then the zwitterion (IV) and cation (V) derived from ethylenediamine react to form an amine (III) and a cation (VIII) with (VII) as an intermediate. Both the H bond as in Equation (6) and the ion reaction could contribute to the formation of intermediate VII. The subsequent ion reaction between (VI) and (VIII) would produce CO_2_ and ethylenediamine. In this way, ethylenediamine could function as the catalyst during the circular reaction route and accelerate the rate‐determining step in Figure 2. It has been reported that the ion reactions are more dramatic in subcritical water, where the H bonding and ion solvation are supported [26].

**FIGURE 3:**
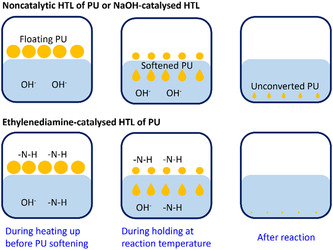
Comparison of the bulk phase behaviour during the non‐catalytic or NaOH‐catalysed HTL and ethylenediamine‐catalysed HTL at different reaction stages.

Besides the molecular level enhancement by the ‐N—H functional group discussed above, another important advantage of the organic base compared to the inorganic base, such as NaOH, is that it could also be present in the vapour phase (Figure 3), especially when the PU foam has not been fully softened and transferred into the liquid phase to have a good contact with the base solution. As PU has low density, it will normally float above the liquid phase before the thermal degradation or transition into lower‐molecular‐weight species of the bulk solid phase. Ethylenediamine has a boiling point at 116.9°C and is very soluble in H_2_O. The ethylenediamine in the vapour phase could accelerate the breakdown of PU, along with that in the liquid phase. On the other hand, the ethylenediamine‐catalysed hydrolysis reaction could decrease the molecular weight of PU. In contrast, the high boiling point of benzylamine (185°C) and dibenzylamine (300°C) could also explain their low catalytic activity for the HTL of PU.

#### 3.1.2 Improved TDA Yield by Alkyl Amine

Typically, 4 g PU together with 100 g water in the presence or absence of 0.2 g catalyst was loaded into an argon‐purged autoclave, initially at atmospheric pressure, and subsequently heated to 180°C and held at self‐generated pressure (e.g. 10 bar) for 30 min. The product is generally composed of a gas phase, an aqueous phase and a paste/solid phase on the reactor wall or bottom. After cooling down, the gas, liquid and solid phase products were collected for further analysis. The liquid, solid and gas yields were 94.7%, 2.8%, and 1.4%, respectively, for the HTL of PU1 at 180°C and 30 min. The gas phase product is mainly composed of CO_2_ based on the GC‐FID/TCD (Supporting information, Figure S4). The CO_2_ is mainly produced from the breakdown of the urethane group. The solid/paste phase product is taken as the unreacted PU even though it may have some oligomers or monomers in it. The produced monomers, especially TDA, are mainly dispersed in the aqueous phase as the solubility of TDA is 7.74 g/L [27]. The TDA was quantified by a GC–MS.

**FIGURE 4:**
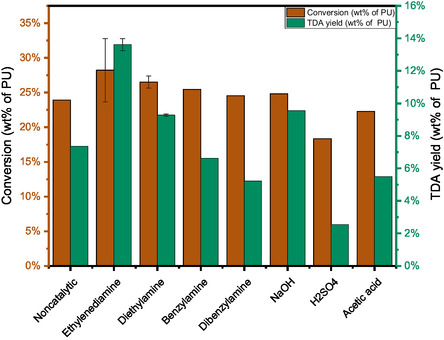
Effect of catalysts on the PU conversion and TDA yield (wt% of reactant PU) (Conditions: 4 g PU1, 100 g H_2_O, 180°C, 30 min, catalyst loading: 5 wt% based on PU).

To make the reaction conditions milder and the decomposition more selective, various kinds of catalysts, such as inorganic and/or organic bases and acids, were tested for the HTL along with the non‐catalytic HTL in the subcritical water (180°C, self‐generated pressure at 10 bar). 180°C was selected so that the performance of various catalysts could be compared at a low conversion. As it is shown in Figure 4, the effects of catalysts on PU conversion are less significant, with the conversion being all around 25 wt%. This indicates that 25 wt% of the PU substrate could be converted under 180°C and 30 min. This agrees with the TGA analysis in Figure S1a (supporting information) that the 28 wt% of PU could be pyrolysed at 275°C. Although a direct comparison with pyrolysis (TGA) data is not strictly appropriate due to the distinct reaction mechanisms and media involved, the reference is included here merely to indicate that HTL achieves a comparable degree of polymer conversion (25 wt% at 180°C) at a substantially lower temperature. This highlights the strong promoting effect of hot compressed water on hydrolytic depolymerisation. More importantly, ethylenediamine features the highest TDA yield of 13.6 wt%. About half of the consumed PU substrate (28.2 wt%) is converted into TDA at 180°C with the selectivity being 48.3 wt%. We propose that ethylenediamine, as an organic base and a nucleophilic agent, is able to initiate the breakdown of PU polymer. The ethylenediamine‐catalysed HTL of PU1 was repeated twice and the standard deviation for the TDA yield was 0.4%.

Compared with equimolar amount of inorganic base NaOH, the organic base ethylenediamine is more effective in terms of PU conversion and TDA yield. For instance, the TDA yield of ethylenediamine catalysed HTL is 13.6%, higher than 9.5% of NaOH at 180°C. Apparently, the basicity is not the only factor determining the catalytic performance, as NaOH has stronger basicity than ethylenediamine. The dissociation equilibrium pK_b_ of NaOH is 0.2 [28], lower than that of an organic base (ethylenediamine pK_b_ = 4.1).

Similarly, H_2_SO_4_ and acetic acid were employed as the inorganic and organic acid, respectively and neither acid improved the conversion. The TDA yield is even reduced to 2.6 wt% for H_2_SO_4_, probably due to the neutralisation reaction with TDA [29]. In addition, acetic acid barely has catalysis effects compared to the non‐catalytic HTL. The decreased TDA yield is consistent with a previous report that the conversion of PU in the presence of acid, such as during acidolysis, could inhibit the formation of amine [10]. This also proves the base is more effective than acid for the HTL of PU. Therefore, compared to inorganic bases and acids, ethylenediamine has the advantage of high activity and selectivity.

To gain more insight into the catalysis mechanism of organic bases, we also used other organic bases. Diethylamine as a monoamine was utilised to catalyse the HTL of PU1 under the same conditions, with the TDA yield being 9.3 wt%, which is between the ethylenediamine‐catalysed and non‐catalytic HTL. Compared to ethylenediamine, diethylamine is less prone to hydrogen bonding in the first adsorption step and thus the subsequent interaction with the PU fractions. To confirm the interaction between PU and the amine, benzylamine and dibenzylamine were utilised as the catalyst and negligible catalysis effects were observed. This is because benzylamine and dibenzylamine have lower solubility in H_2_O than ethylenediamine, which makes their interaction with PU or its degraded intermediates less feasible.

The NMR of paste phase product from both non‐catalytic and catalytic HTL of PU were measured as shown in Figure S5 (supporting information). All the paste products have similar shifts, confirming the overall reaction route in Figure 1. Similarly, the FT‐IR spectra of both paste and H_2_O phase products from non‐catalytic and ethylenediamine catalysed HTL of PU1 are shown in Figure S6 (supporting information). The similar spectra in both paste and aqueous phases also prove the similar product distribution and thus confirm the above mechanism.

#### 3.1.3 HTL at Higher Temperature and Longer Reaction Time

**FIGURE 5:**
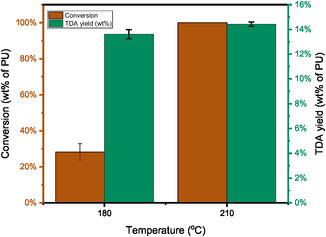
Effects of temperature on the PU1 conversion and TDA yield (wt% of PU) (Conditions: 4 g PU1, 100 g H_2_O, 30 min, ethylenediamine catalyst loading: 5 wt% based on PU).

**FIGURE 6:**
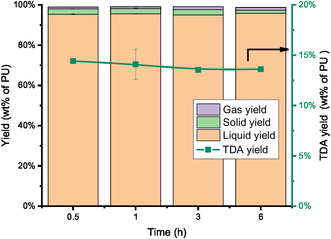
Effects of time on the mass balance and TDA yield (wt% of PU) (Conditions: 4 g PU1, 100 g H_2_O, 210°C, ethylenediamine catalyst loading: 5 wt% based on PU).

To fully convert the PU, the HTL of PU1 was also conducted at 210°C. Interestingly, PU1 has a quantitative conversion even under non‐catalytic conditions. However, the increase of conversion does not improve the TDA yield as shown in Figure 5. In addition, the product distributions at 210°C for both non‐catalytic and ethylenediamine‐catalysed HTL appear similar, as indicated by the overlapping FT‐IR spectra shown in Figure S7 (Supporting information). The comparable presence and relative intensities of characteristic peaks suggest that the PU backbone undergoes a similar initial depolymerisation pathway under both conditions. This indicates that, while ethylenediamine accelerates the reaction, it does not fundamentally alter the primary cleavage mechanism. Under these conditions, the maximum TDA yield for the HTL of PU1 was 14.4 wt%, demonstrating the efficiency of the catalytic process without changing the overall depolymerisation profile. To further confirm this, the reaction time was extended to up to 6 h and the TDA yield is stable around 14 wt% as shown in Figure 6. The highest TDA yield is similar to the reported value in literature [12]. Based on the GC–MS, the other components besides TDA are mainly cyclic amines such as 1,2,3,4‐tetrahydro‐2,3‐dimethylquinoxaline (Supporting information, Figure S8).

### 3.2 Effects of Ethylenediamine on the HTL of Various PU

#### 3.2.1 Validation Using Different PU

**FIGURE 7:**
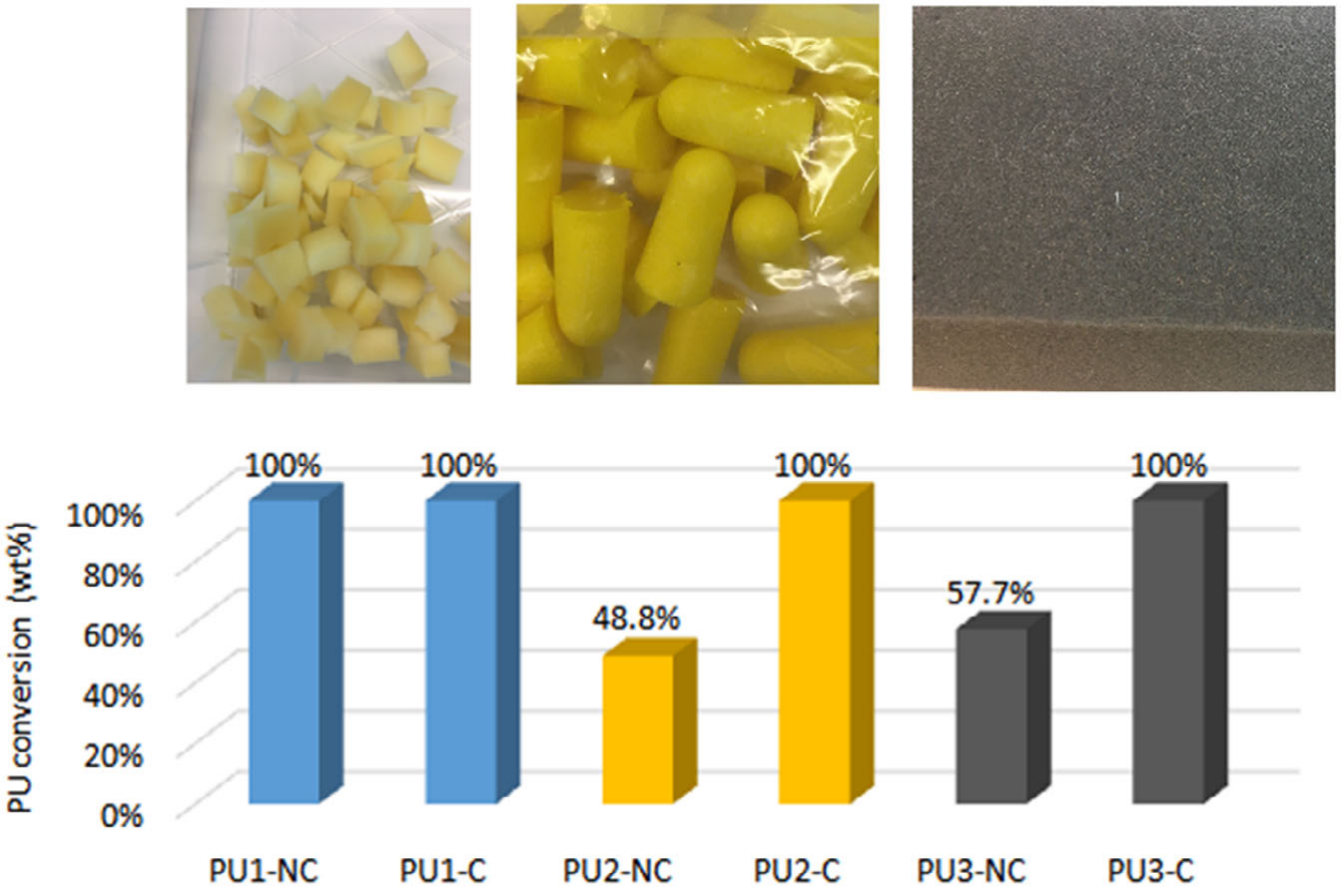
Conversion of the (non)catalytic HTL of various PU (4 g PU in 100 g H_2_O in 450 mL autoclave, 210°C, reaction time: 30 min, 400 rpm, self‐generated pressure, NC: non‐catalytic and C: ethylenediamine‐catalysed).

To validate the concept, various waste PU polymers were hydrothermally liquefied both non‐catalytically and catalytically at 210°C with the conversion shown in Figure 7. The catalytic PU conversion into monomers was significantly higher compared to the non‐catalytic HTL, especially for PU2 and PU3, which indicates that ethylenediamine functions as an effective catalyst to promote the depolymerisation of waste PU. However, the TDA yield is improved only slightly when using ethylenediamine as the catalyst, as shown in Figure S9 (supporting information). The reason is that 210°C and 30 min are high enough to convert the TDI‐derived PU fraction. Based on the TGA analysis in Figure S1 (supporting information), we can see that the PU2 from the ear plug is nearly composed of one kind of PU. To illustrate the catalytic effects of ethylenediamine more clearly, the characterisation of PU2 HTL products will be discussed in detail as follows.

#### 3.2.2 Comparison of Non‐Catalytic and Catalytic HTL of PU2

When the HTL reaction was done non‐catalytically, liquid and paste fractions were obtained, whereas with catalyst, only a single phase was obtained. For PU2, the GC–MS of the liquid and paste phase products from non‐catalytic HTL as shown in Figure S10a,b indicates that the soluble fractions are mainly TDA. There is also a small amount of 4,4′‐methylenedianiline (MDA) present in the product. Only the THF‐dissolvable part was analysed for the paste fraction. Most of the aqueous phase is composed of H_2_O, which is not given in Figure S10a due to the avoidance of the solvent peak in the first 4 min in the retention time. The species are determined by comparing with the NIST database 2020. The GC–MS of the one single phase product from the ethylenediamine‐catalysed HTL is shown in Figure S10c, which indicates the TDA is the main component. PU3 has a similar product distribution to PU2 as it is shown in the Figure S11 in supporting information. The relatively simple product distribution indicates that the catalytic HTL is an effective chemical recycling method because it can convert PU into the monomers or their derivates. In another word, the catalytic HTL could crack the urethane groups (–HN–COO–) while keeping the other C—C bonds intact, indicating that the catalyst is selective to the urethane groups.

The liquid phase products from HTL of PU mainly include amines, e.g. TDA for toluene‐2,4‐diisocyanate (TDI) and MDA from 4,4′‐methylene diphenyl diisocyanate (MDI). The MDA and TDA are the desired monomers from the decomposition of PU. The TDI and MDI are typical diisocyanates for the synthesis of PU. It can be concluded that ethylenediamine could work for both MDI and TDI‐derived PU.

During the non‐catalytic HTL of PU in a solid–liquid system, the first step is to soften the PU foam so that PU can have good contact with the liquid H_2_O because the low density of PU would generally make most of the PU float on the liquid. For example, the PU1 used in this study has a melting point around 180°C (Figure S1a, supporting information). The softened PU falls into the liquid phase in form of flowable paste which continues the chemical decomposition gradually. For the ethylenediamine‐catalysed HTL, besides the liquid phase, the depolymerisation might also occur in the vapour phase when the H_2_O and ethylenediamine solution is in vapour‐liquid equilibrium in the pressurised reactor, which could explain the accelerated conversion in Figure 7. This indicates that the decomposition of PU under hydrothermal conditions progresses in a gradual way, which means from larger polymer to oligomer and finally to small molecules such as amine.

**FIGURE 8:**
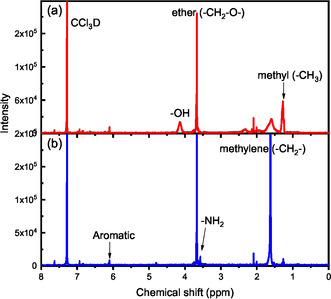
^1^H NMR of products from the catalytic HTL of PU2: (a) paste phase products of non‐catalytic HTL of PU2 and[jls‐backslash] (b) one single liquid phase product from PU2 catalysed by ethylenediamine at 210°C and 30 min.

**FIGURE 9:**
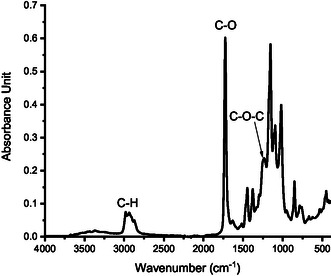
FT‐IR spectra of the paste phase products of non‐catalytic HTL of PU2 at 210°C and 30 min.

The ^1^H NMR of the paste phase product from HTL of PU2 is shown in Figure 8a. All the paste phase products could be dissolved in the CCl_3_D solvent. The identification of the NMR peaks agrees with the species determined by GC–MS in Figure S10b. The amine presence could be supported by the chemical shift at 3.57 ppm from the primary amine and 6.1 ppm from the aromatic hydrogen. Besides being derived from the small monomers and their derivatives, the primary amine may be at the terminal of the oligomer chain. The hydroxyl proton and aliphatic hydrogen mainly form the polyols. Similarly, these functional groups, such as C—O and N—H could also be verified by the FT‐IR spectra of the paste phase product from HTL of PU1 as illustrated in Figure 9 [30, 31]. In addition, with FT‐IR in Figure S12 compared to that of the raw PU in Figure S2, the overall intensities of N—H peak increase, whereas C—O peak decreases significantly, which is caused by the cleavage of urethane bonds and the formation of amine.

## 4 Conclusions

In this study, we investigated a variety of catalysts to promote the HTL of PU and found that ethylenediamine as an organic catalyst is more effective for different waste PU in terms of TDA yield and PU conversion, compared to the inorganic base or acid, because the N—H functional group of ethylenediamine could accelerate the cracking in both vapour and liquid phase selectively. Moreover, the diamine is more effective than monoamines such as diethylamine and benzylamine.

Mechanistically, the ester group in the urethane linkage is the most readily cleaved, whereas C—N bond cleavage represents the rate‐determining step in non‐catalytic or NaOH‐catalysed reactions. Generally, PU first undergoes hydrolysis to form carbamic acid and polyol, followed by further hydrolysis to generate amines and CO_2_. Ethylenediamine facilitates the decomposition of carbamic acid into CO_2_ and amines via ionic interactions with the zwitterion formed during tautomerism, thus functioning as an efficient catalyst for HTL. Under the studied conditions, ethylenediamine achieved a TDA yield of 13.6 wt% with 28.2% PU conversion at 180°C, while the maximum TDA yield reached 14.4 wt% for PU1 at 210°C with 5 wt% ethylenediamine.

## Supporting Information

Additional supporting information can be found online in the Supporting Information section. **Supporting Fig. S1:** TGA and DTG of (a) PU1 from mattress, (b) PU2 from ear plug and (c) PU3 from black packing foam. **Supporting Fig. S2:** FT‐IR of (a) PU1 (mattress), (b) PU2 (yellow ear plug), and (c) PU3 (black package foam). **Supporting Fig. S3:** Calibration line between TDA concentration (wt%) and the area of extracted ion chromatograms (ion=122). **Supporting Fig. S4:** GC‐FID/TCD of gas phase products (4 g PU in 100 g H_2_O in 450 mL autoclave, 180 °C, reaction time: 30 min, 400 rpm, self‐generated pressure). **Supporting Fig. S5:** ^1^H‐NMR of paste phase product from noncatalytic and catalytic HTL of PU1 (Conditions: 4 g PU1, 100 g H_2_O, 180 °C, 30 min, catalyst loading: 5 wt% based on PU). **Supporting Fig. S6:** FT‐IR spectra of product from noncatalytic and ethylenediamine‐catalysed HTL of PU1 (Conditions: 4 g PU1, 100 g H_2_O, 180 °C, 30 min, catalyst loading: 5 wt% based on PU). **Supporting Fig. S7:** FT‐IR spectra of product from noncatalytic and ethylenediamine‐catalysed HTL of PU1 at 210 °C (Conditions: 4 g PU1, 100 g H_2_O, 210 °C, 30 min, catalyst loading: 5 wt% based on PU). **Supporting Fig. S8:** GC‐MS of both aqueous and paste phase product (Conditions: 4 g PU1, 100 g H_2_O, 210 °C, 6 h, catalyst loading: 5 wt% based on PU). **Supporting Fig. S9:** TDA yield (wt% of PU) from the HTL of various PU (4 g PU in 100 g H_2_O in 450 mL autoclave, 210 °C, reaction time: 30 min, 400 rpm, self‐generated pressure, NC: noncatalytic and C: ethylenediamine‐catalysed). **Supporting Fig. S10:** GC‐MS of products from the catalytic HTL of PU2: (a) liquid phase and (b) paste phase products of PU2, (c) one single liquid phase product from PU2 catalysed by ethylenediamine at 210 °C and 30 min. **Supporting Fig. S11:** GC‐MS of products from the catalytic HTL of PU3 catalysed by ethylenediamine at 210 °C and 30 min: aqueous phase (a) and paste phase (b) fractions. **Supporting Fig. S12:** FT‐IR spectra of products from the HTL of PU: (a) liquid phase products of noncatalytic HTL of PU2 and (b) one single liquid phase product from PU2 catalysed by ethylenediamine at 210 °C and 30 min.

## Conflicts of Interest

The authors declare no conflicts of interest.

## Supporting information

Supplementary Material

## Data Availability

The data that support the findings of this study are available in the supplementary material of this article.
