# Supplementary material for: Recycling of Polyurethane Waste: Facile Hydrothermal Conversion Using Acidic and Basic Additives
Source: ChemSusChem. 2026 Jan 5;19(1):e202502372. doi: 10.1002/cssc.202502372 (PMC12766875; doi:10.1002/cssc.202502372)
Supplement: Supplementary file 1 — Supplementary Material [file CSSC-19-e202502372-s001.pdf]

Supporting information

Recycling of Polyurethane Waste: Facile Hydrothermal Conversion using Acidic and Basic Additives

Hongqi Wang, Himanshu Gupta, N. Raveendran Shiju*

Catalysis Engineering Group, Van 't Hoff Institute for Molecular Sciences, University of Amsterdam, 1090 GD Amsterdam, The Netherlands

* Email: n.r.shiju@uva.nl

S1. PU feedstock characterisation

To have an understanding of the decomposition behaviour of PU during HTL, the PU feedstocks were analysed using TGA as shown in Fig.S1.

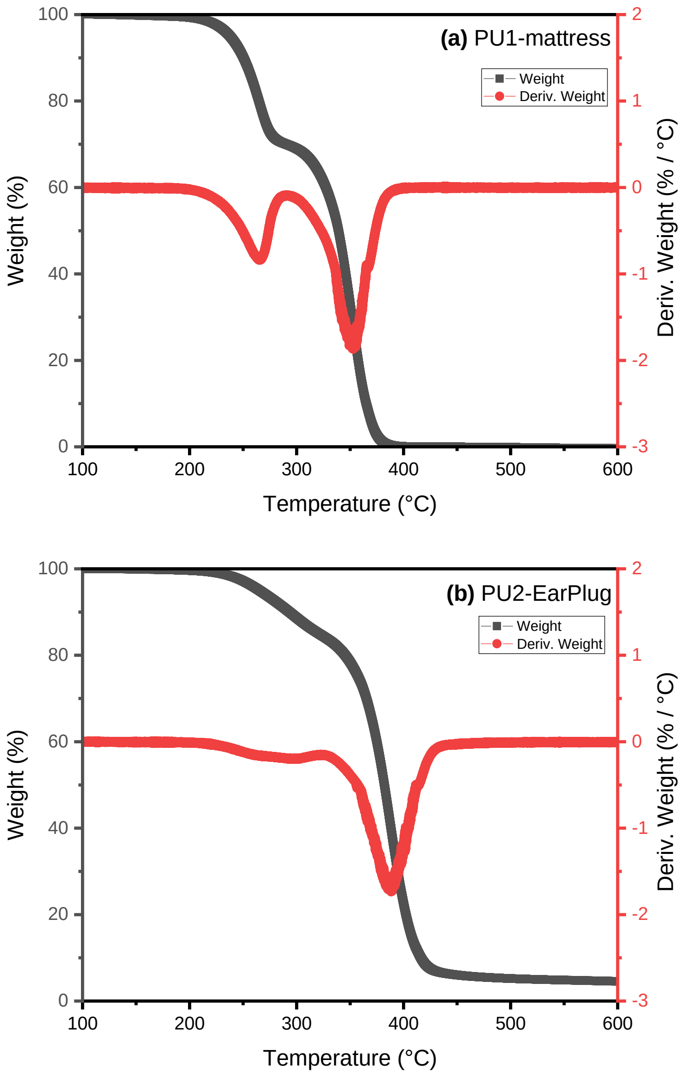

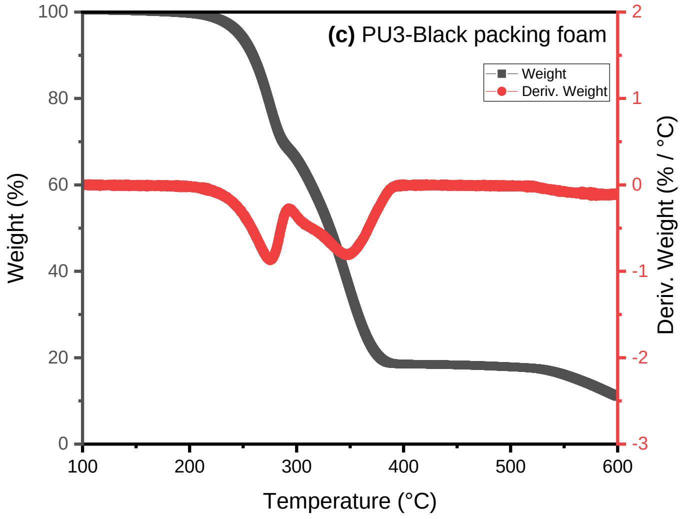

Fig.S1. TGA and DTG of (a) PU1 from mattress, (b) PU2 from ear plug and (c) PU3 from black packing foam.

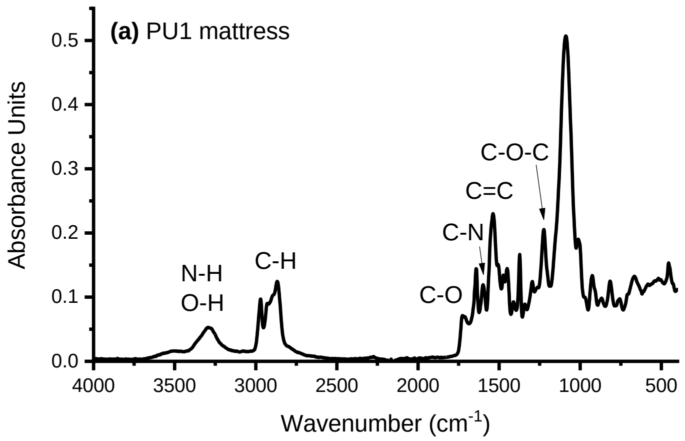

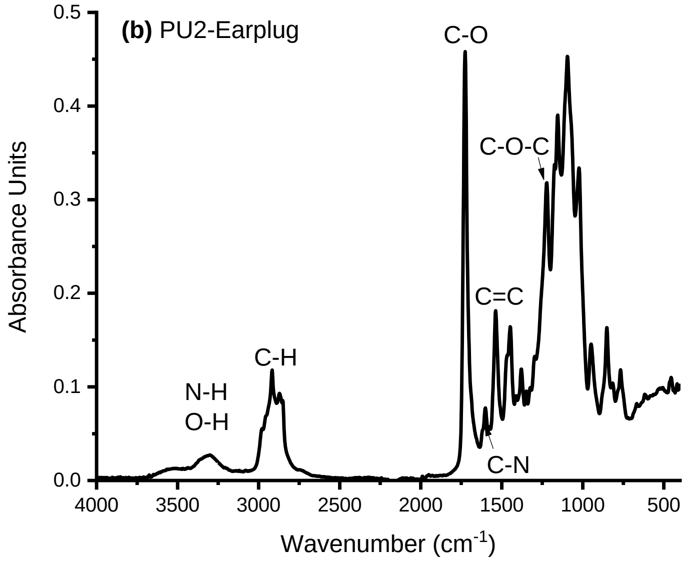

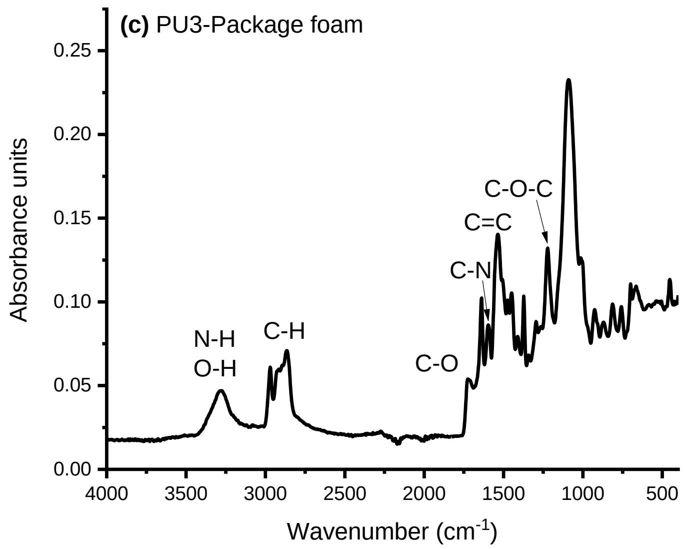

Fig.S2. FT-IR of (a) PU1 (mattress), (b) PU2 (yellow ear plug), and (c) PU3 (black package foam).

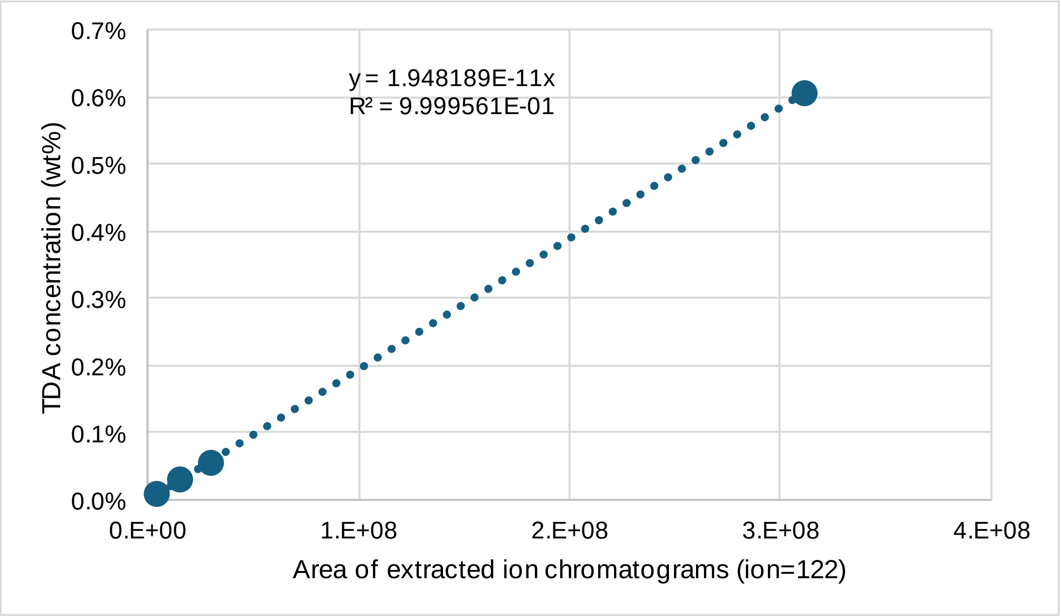

Fig.S3. Calibration line between TDA concentration (wt%) and the area of extracted ion chromatograms (ion=122).

S2. Product analysis

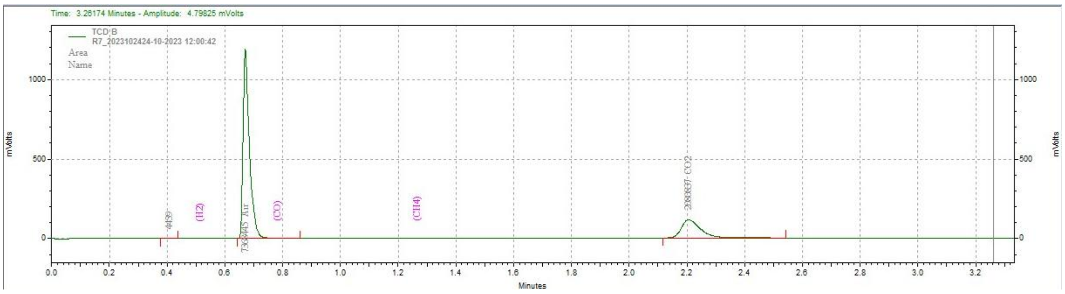

Fig.S4. GC-FID/TCD of gas phase products (4 g PU in 100 g H_2O in 450 mL autoclave, 180 °C, reaction time: 30 min, 400 rpm, self-generated pressure).

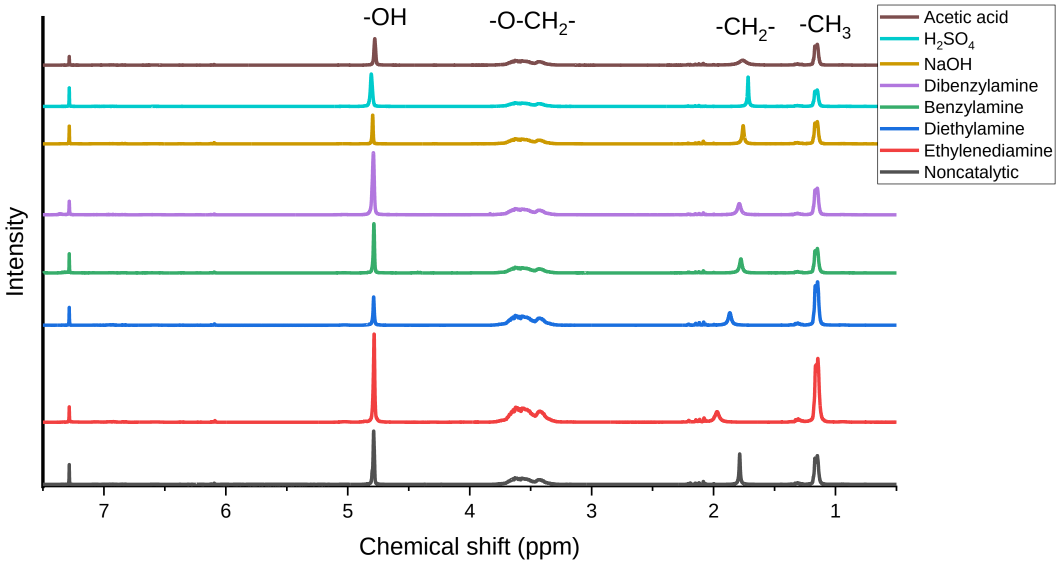

Fig.S5. ^{1}H-NMR of paste phase product from noncatalytic and catalytic HTL of PU1 (Conditions: 4 g PU1, 100 g H_2O, 180 °C, 30 min, catalyst loading: 5 wt% based on PU).

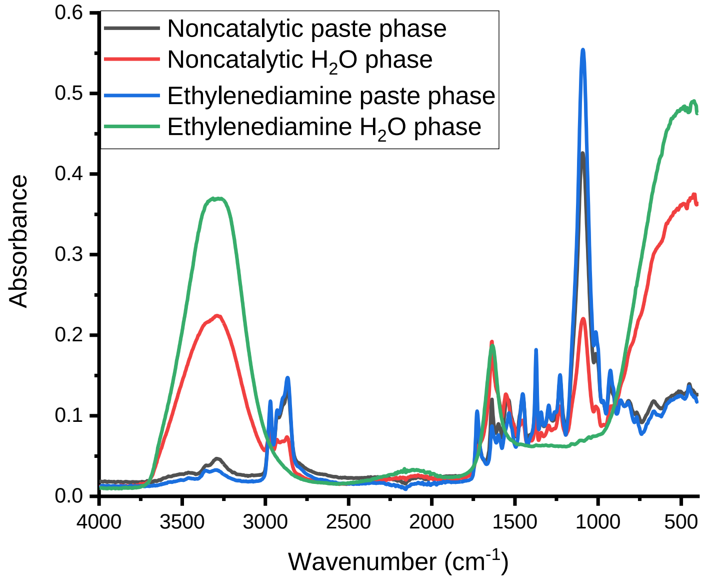

Fig.S6. FT-IR spectra of product from noncatalytic and ethylenediamine-catalysed HTL of PU1 (Conditions: 4 g PU1, 100 g H_2O, 180 ^oC, 30 min, catalyst loading: 5 wt% based on PU).

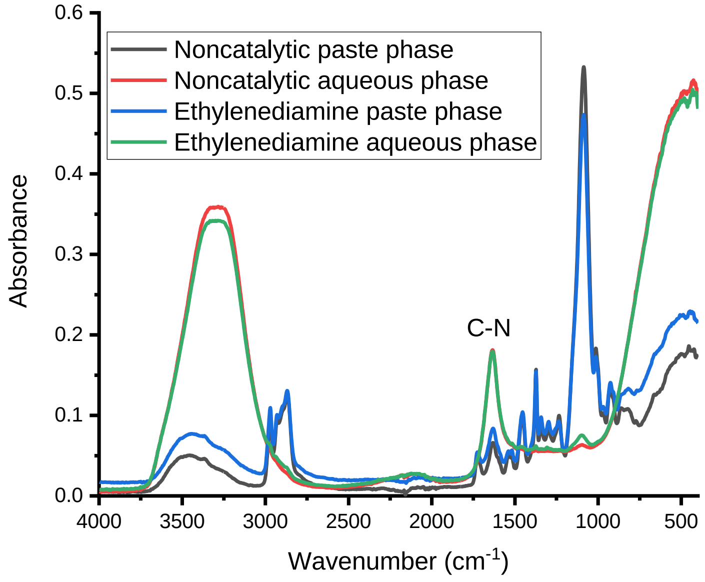

Fig.S7. FT-IR spectra of product from noncatalytic and ethylenediamine-catalysed HTL of PU1 at 210 ^oC (Conditions: 4 g PU1, 100 g H_2O, 210 ^oC, 30 min, catalyst loading: 5 wt% based on PU).

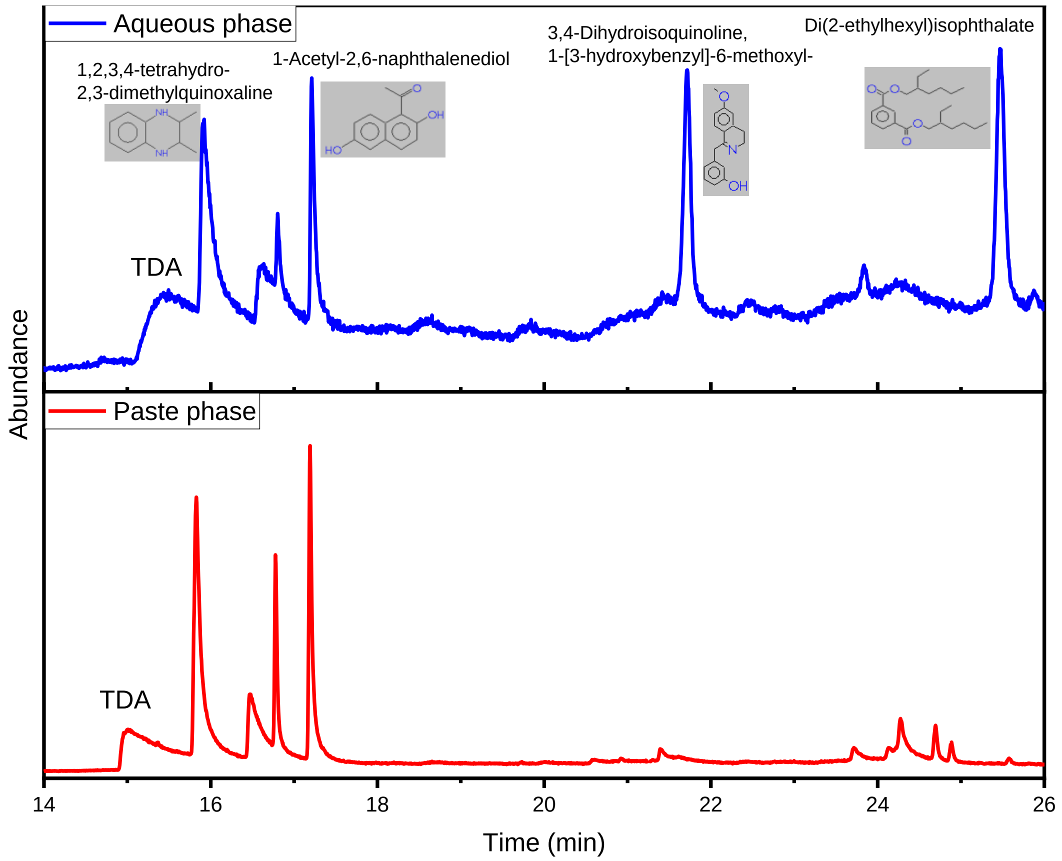

Fig.S8. GC-MS of both aqueous and paste phase product (Conditions: 4 g PU1, 100 g H_2O, 210 ^oC, 6 h, catalyst loading: 5 wt% based on PU).

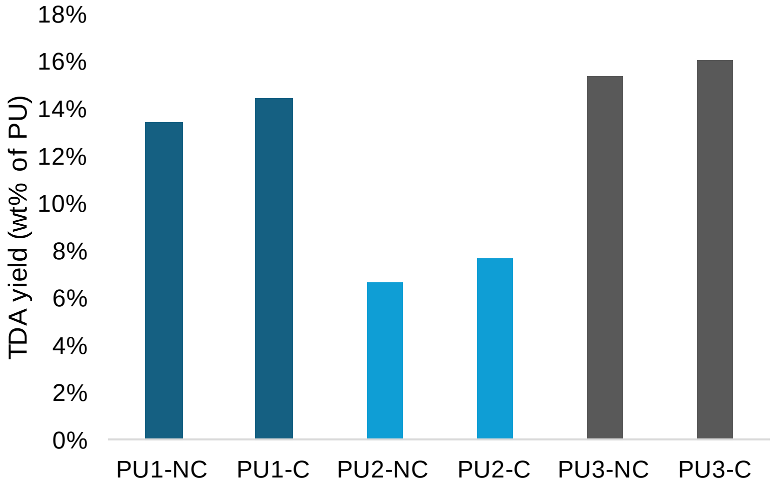

Fig.S9. TDA yield (wt% of PU) from the HTL of various PU (4 g PU in 100 g H_2O in 450 mL autoclave, 210 ^oC, reaction time: 30 min, 400 rpm, self-generated pressure, NC: noncatalytic and C: ethylenediamine-catalysed).

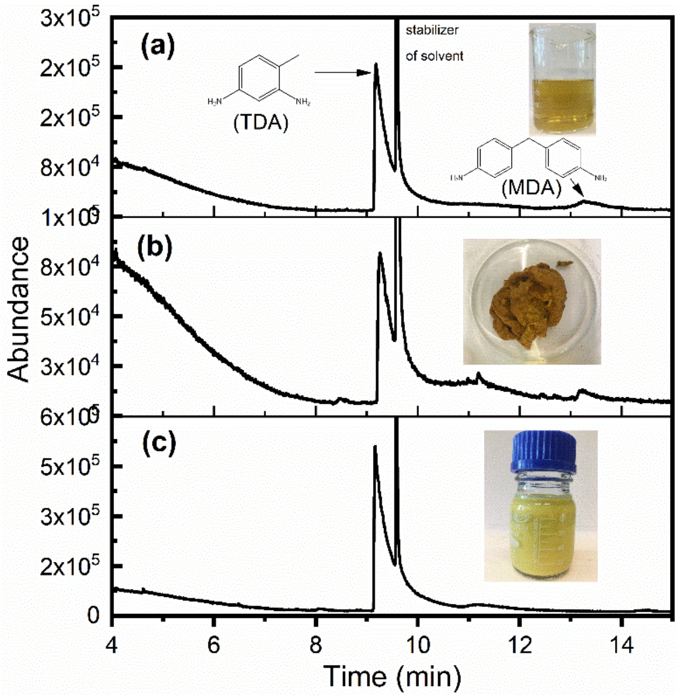

Fig.S10. GC-MS of products from the catalytic HTL of PU2: (a) liquid phase and (b) paste phase products of PU2, (c) one single liquid phase product from PU2 catalysed by ethylenediamine at 210 ^{o}C and 30 min.

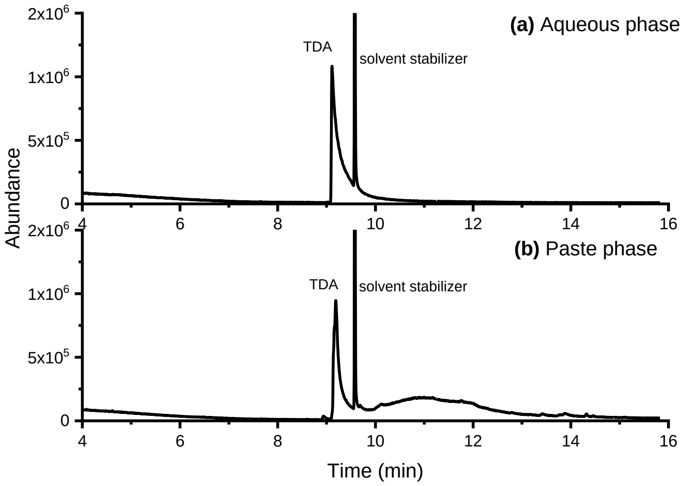

Fig.S11. GC-MS of products from the catalytic HTL of PU3 catalysed by ethylenediamine at 210 ^{o}C and 30 min: aqueous phase (a) and paste phase (b) fractions.

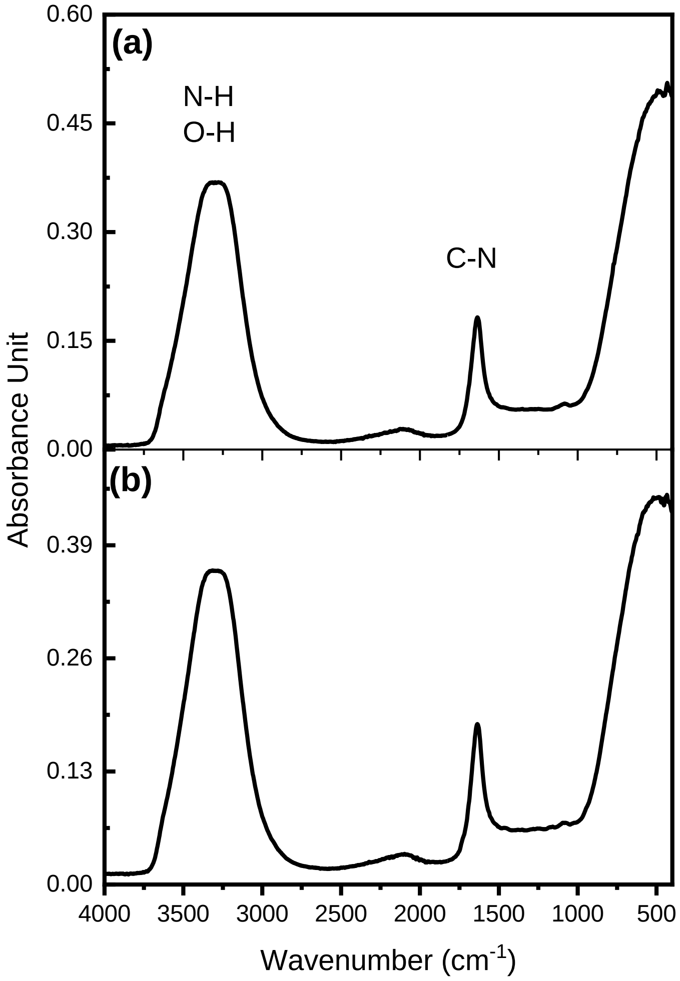

Fig.S12. FT-IR spectra of products from the HTL of PU: (a) liquid phase products of noncatalytic HTL of PU2 and (b) one single liquid phase product from PU2 catalysed by ethylenediamine at 210 ^{o}C and 30 min.
